# Discrimination of Motor Imagery-Induced EEG Patterns in Patients with Complete Spinal Cord Injury

**DOI:** 10.1155/2009/104180

**Published:** 2009-04-29

**Authors:** G. Pfurtscheller, P. Linortner, R. Winkler, G. Korisek, G. Müller-Putz

**Affiliations:** ^1^Laboratory of Brain-Computer Interfaces, Institute for Knowledge Discovery, Graz University of Technology, Krenngasse 37, 8010 Graz, Austria; ^2^Rehabilitation Clinic Tobelbad, Krenngasse 37, Dr.-Georg-Neubauer-Straße 6, 144 Tobelbad, Austria

## Abstract

EEG-based discrimination between different motor imagery states has been subject of a number of studies in healthy subjects. We investigated the EEG of 15 patients with complete spinal cord injury during imagined right hand, left hand, and feet movements. In detail we studied pair-wise discrimination functions between the 3 types of motor imagery. The following classification accuracies (mean ± SD) were obtained: left versus right hand 65.03% ± 8.52, left hand versus feet 68.19% ± 11.08, and right hand versus feet 65.05% ± 9.25. In 5 out of 8 paralegic patients, the discrimination accuracy was greater than 70% but in only 1 out of 7 tetraplagic patients. The present findings provide evidence that in the majority of paraplegic patients an EEG-based BCI could achieve satisfied results. In tetraplegic patients, however, it is expected that extensive training-sessions are necessary to achieve a good BCI performance at least in some subjects.

## 1. Introduction

Functional magnetic resonance imaging (fMRI) and EEG studies have shown that executed and imagined movement activates overlapping and/or similar neural networks in primary motor and related areas [1–3]. This equivalence of motor execution and motor imagery in relation to cortical activation is one prerequisite for the restoration of motor functions in para- and/or tetraplegic patients using a brain-computer interface (BCI; [4, 5]). Whether patients with complete spinal cord injury (SCI) are able to control their brain oscillations reliable and safe through imagined limb movements and operate herewith a BCI is however still an open question.

Sensorimotor rhythms such as mu and central beta oscillations can be modified by executed and imagined movement [6–10]. By using multichannel EEG recordings and applying pair-wise discrimination functions to the EEG signals it is possible to discriminate between 3 different types of motor imagery (right or left hand or foot) [11, 12]. In this study we addressed the following questions: (i) is it possible to discriminate pair-wise between 3 motor imagery-related EEG patterns (right hand, left hand, and feet) in patients with complete spinal cord injury and (ii) is this discrimination different for paraplegic and tetraplegic patients. When a reliable detection of imagery-related brain states in ongoing EEG is possible the BCI output signal can be used to control, for example, a neuroprosthesis [5].

## 2. Methods

### 2.1. Subjects and Experimental Task

The patient group consisted of 15 patients (four females and eleven males) aged from 16 to 64 years (M = 41 years, SD = 14.50). All patients suffered from a complete sensor and motor paralysis at ASIA level C5 to T12 after a traumatic SCI between 1.6 months and 32.9 years prior to the measurements. Seven patients were tetraplegic, and eight patients were paraplegic. Information on the patients is summarized in Table 1.

Measurements were carried out at the Rehabilitation Clinic Tobelbad (Austria). The experiment was divided into 6–8 runs (depending on the physical condition of the patient), each consisting of 30 trials of three different motor imagery tasks (10 trials each). Between those runs participants could (and were encouraged to) take short breaks for recovery and in order to avoid fatigue.

Each trial began with the presentation of a fixation cross at the centre of the monitor, followed by a short warning tone at second 2. At second 3, an arrow pointing randomly left, right, or down, representing one of three different motor imagery tasks (left hand (L), right hand (R) and both feet (F), resp.), appeared on the screen for 1.25 seconds, additionally to the fixation cross. The fixation cross remained displayed on the screen until the end of the trial at second 8, indicating that the imagination still had to be performed. This implies a motor imagery lasting for 5 seconds was required. After that, a blank screen was presented until the beginning of the next trial. This intertrial period varied randomly between 0.5 and 2.5 seconds.

**Figure 1:**
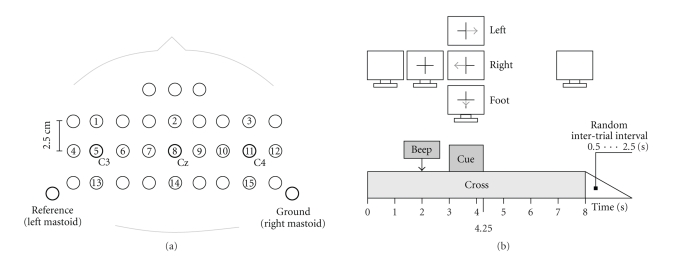
(a) Electrode positions. (b) Timing and experimental paradigm.

Timing and experimental paradigm are displayed in Figure 1(b).

### 2.2. EEG Recording

Continuous EEG signals were recorded from a grid of fifteen sintered Ag/AgCl ring electrodes (Easycap, Germany) that were mounted orthogonally in both, horizontal and vertical directions, over the electrode positions C3, Cz, and C4 (according to the international 10–20 electrode system, cf. Figure 1(a)). The closely spaced interelectrode distance was 2.5 cm. All electrodes were referenced to the left mastoid. The ground electrode was mounted at the right mastoid. Impedances were kept below 5 kOhm. For monopolar EEG derivation a portable amplifier (g.tec, Graz, Austria) was used. Signals were digitized at 256 Hz and bandpass filtered between 0.5 and 100 Hz. Sensitivity was set to 100 *μ*V and a notch filter at 50 Hz was used.

### 2.3. Data Analysis

The method of Common Spatial Patterns (CSP) and Fischer's linear discriminant analysis (LDA) classifier were used to discriminate between any 2 classes. The CSP-method projects multichannel EEG data into a low-dimensional spatial subspace in such a way that the variances of the filtered time series are optimal for discrimination. The projection matrix, consisting of the weights of the EEG channels, is sorted in descending order of the eigenvalues. Before applying CSP and LDA, a fully automated method for reducing EOG artifacts was applied on the data. Then, the EEG recordings were visually inspected for remaining EOG and EMG artifacts and filtered between 8–30 Hz. To get a good generalization of the classifier a 10 × 10 cross-validation procedure was adopted. The EEG data from each trial was divided into time segments of 1 s overlapping by half of their length. For further details see [11, 14].

### 2.4. Calculation of Time-Frequency Maps

To enhance local oscillations, orthogonal source derivations (Laplacian) were calculated [15]. After triggering the data, trials of 10 s duration were obtained including 3 seconds before the cue. The quantification of ERD/ERS was carried out in four steps: band pass filtering of each trial, squaring of samples (with smoothing) and subsequent averaging over trials and over sample points. The ERD/ERS is defined as the percentage power decrease (ERD) or power increase (ERS) in relation to a one-second reference interval (0.5–1.5 seconds ) before the warning tone [3]. ERD/ERS values corresponding to 2-Hz frequency bands ranging from 6–18 Hz (with an overlap of 1 Hz) and 4-Hz frequency bands ranging from 18–38 Hz (with an overlap of 2 Hz) were calculated. All values for one EEG channel were subsequently used to construct time-frequency maps (ERD/ERS maps). The statistical significance of the ERD/ERS values was verified by applying a *t*-percentile bootstrap statistic to calculate confidence intervals with *α* = 0.05.

### 2.5. Statistical Analysis

An ANOVA was computed in order to examine whether paraplegic versus tetraplegic patients differ regarding reached classification accuracy. This ANOVA consisted of the between-subject variable SCI (2 levels: paraplegics and tetraplegics) and the within-subject variable ACCURACY (3 levels: left hand versus right hand, left hand versus feet and right hand versus feet).

## 3. Results

**Figure 2:**
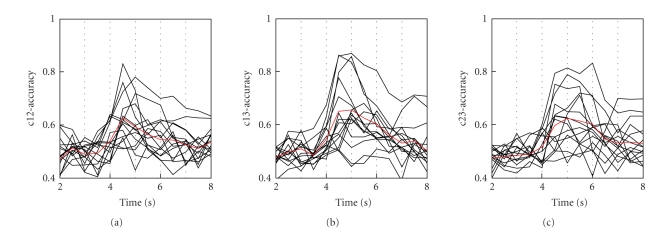
Discrimination time courses for a length of 5 s after cue onset. The onset of cue presentation is at second 3. Data from all 15 patients and all 3 brain states are displayed: right versus left hand MI (left panel), left hand versus feet MI (middle panel), and right hand versus feet MI (right panel).

The power of discrimination between two different brain states is indicated by the classification accuracy of single EEG trials analysed within 1-second time windows. The discrimination time courses for epochs of 6 seconds (with 1 second prior to cue-onset) for all task combinations (right versus left hand, left hand versus feet, and right hand versus feet) are shown in Figure 2. The maximal classification accuracies of the first peak together with the corresponding latencies, measured from cue onset are summarized in Table 2. The mean accuracy of all subjects (±SD) was 65.03% ± 8.51 (left versus right hand MI), 68.18% ± 11.08 (left hand versus feet MI) , and 65.05% ± 9.25 (right hand versus feet MI), respectively. (See Table 3 for the mean accuracy of paraplegic versus tetraplegic patients.)

In the tetraplegic patient group only one out of seven tetraplegics had an accuracy >70% while from the paraplegics five out of 8 reached a classification accuracy >70%. An accuracy of 70% is the border, where control can be possible [16]. In the majority of participants, feet motor imagery was involved in the best discrimination between two brain states (see Figure 2 and Table 2).

**Figure 3:**
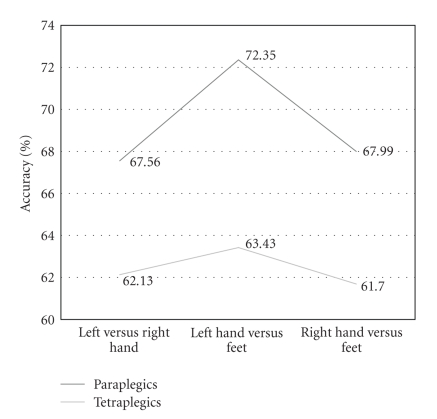
Two-class classification accuracy for paraplegic and tetraplegic patients.

The results of the ANOVA show that the main effect ACCURACY is insignificant. Paraplegic patients (M = 69.3%) do not differ from tetraplegic patients (M = 62.41%), F(1,13) = 530.292, *p* = .151. Furthermore, no significant effect emerges for the three classification accuracies, left (L) hand versus right (R) hand (M = 64.85%), left (L) hand versus feet (F) (M = 67.89%), and right (R) hand versus feet (F) (M = 64.84%), but a tendency can be seen, F(2,26) = 2.877, *p* = .074 (cf. Figure 3).

**Figure 4:**
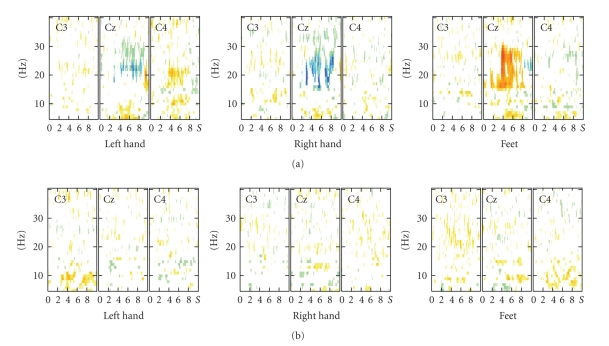
Time-frequency-maps for the three types of motor imagery (left hand, right hand, and feet) computed at electrode positions C3, C_Z_, C4 (Laplacian) exemplarily for (a) a patient with good performance (p02) and (b) a patient with bad performance (p01).

Although the discriminations of any two different brain states were based on the analysis of 15 EEG channels recorded over premotor, motor, and parietal areas, different patterns were found in spatially filtered (Laplacian) recordings over the primary motor areas (electrode positions C3, Cz, and C4). For illustration, time-frequency maps (ERD-maps) of two representative subjects are displayed in Figure 4. In subject P02 (Figure 4(a)) clearly visible is the beta increase (ERS) at Cz during hand MI and the beta decrease (ERD) at Cz during feet MI. No clear EEG reactivity patterns can be recognized in subject P01 (Figure 4(b)). In P02 a high classification accuracy was obtained while in P01 no discrimination between the motor imagery states was possible.

## 4. Discussion

In our study, we applied a classification procedure to multichannel, single-trial EEG data recorded during classical brain-computer interface training sessions with 3-motor imagery tasks: right-hand, left-hand, and feet movement [17, 18]. One method suitable for studying temporal aspects of brain activation using multichannel EEG recordings consists in computing common spatial patterns (CSPs) [14]. This CSP-method leads to spatial filters that are optimal in the sense that they extract signals which maximally discriminate between any 2 conditions. A subsequent linear classification of these extracted signals results in a good recognition rate. With the CSP-method it is possible to study the separability of EEG patterns associated with 2-motor imagery states with a high time resolution.

The discrimination time courses in the patients with complete spinal cord injury studied were highly variable in its shape and magnitude and started in generally with an initial peak about 1.5 seconds after cue-onset, with a fast increase before and a slow decline thereafter (Figure 2). The great intersubject-variability may be explained by the used mental strategy (e.g., visual versus kinaesthetic motor imagery, [19]), the vividness of the imagery process, the mental effort and other psychological factors as, for example, motivation and attention. Even in one and the same subject the same mental motor imagery strategy can result in completely different EEG reactivity patterns dependent on the degree of imagined effort [20].

The main finding of the present study is that there are distinct EEG patterns in the majority of patients with complete spinal cord injury when they imagine different movements of hands and feet the first time. These patterns are however not very pronounced and the mean classification rate was relatively low around 67%. In contrast, motor imagery in healthy subjects results in clearly discriminable EEG patterns, when 2-motor imagery tasks are compared. Blankertz et al. [17] reported a mean classification accuracy of 88.4% in a so-called calibration session with 3 types of motor imagery (right hand, left hand, and right foot) in untrained healthy subjects. This data are based on 128 EEG channels and CSP analysis. Also with CSP analysis applied to 32 EEG channels mean classification accuracies between 80.0% and 83.3% are reported for left versus right hand MI and hand versus feet MI [12]. In both studies in the majority of subjects the best classification results were achieved when foot MI was involved. One major difference between healthy subjects and patients is very often that patients have very often cramps and/or spasms and therefore a number of muscle artefacts in the EEG (see e.g., Table 1 artefact-free versus total trials).

Of interest is a recently published fMRI study where control subjects and patients had to kinaesthetically imagine movements of their feet [21]. In the paraplegic patient group the primary motor cortex was consistently activated, even to the same degree as during movement execution in the healthy controls. In contrast to this one other study [22] reported inconsistent fMRI activation in the primary motor cortex during self-paced foot motor imagery in complete SCI patients. Of interest is that in the study of Alkadhi et al. [21], a strong positive correlation was found between the vividness scores of motor imagery in paraplegics and the activation (fMRI BOLD signal) in cortical areas including the primary motor cortex and the supplementary motor area (SMA). This can be interpreted that vividness of motor imagery and/or their mental effort plays an important role in cortical activation and is perhaps more intensive in SCI patients than in healthy controls.

One point needs discussion, namely, the slightly higher (but not significant) classification accuracy of hand versus feet MI as compared to right versus left hand MI found in patients but also reported in healthy subjects. This can be interpreted to mean that the EEG patterns induced by feet or foot MI are better discriminable from the brain state associated with either left or right hand MI. One reason for this could be the antagonistic behaviour of the upper mu ERD and ERS during motor imagery known as “focal ERD/surround ERS” [3]. Feet MI results not only in a midcentrally focused mu and/or beta ERD but very often also in a bilateral mu ERS over the hand representation area [23]. These authors reported on a much larger difference in band power changes in the 10–12 Hz frequency band when different (hand versus foot MI) and not homologous limbs (right versus left hand MI) are compared.

In conclusion, we demonstrated that in the majority of paraplegic patients motor imagery induced EEG patterns can be discriminated. From this follows that with a small number of feedback training sessions the separability between motor imagery-related brain states can be reinforced and a good BCI performance can be expected. In tetraplegic patients the situation is less clear. Only in one patient motor imagery-related EEG patterns could be discriminated in the initial training session. Here extensive trainings sessions without and with feedback are necessary to achieve a satisfied BCI performance at least in some patients.

## Figures and Tables

**Table 1:** Patients characteristics.

Patient	Date of birth (year)	Date injury (year)	Duration (months)	ASIA level	Number of trials (artifact-free/total)
P01	1987	2007	3.9	C5 (Tetra)	207/240
P02	1981	2007	5.3	C6 (Tetra)	220/240
P03	1957	1991	192	Th12 (Para)	222/240
P04	1972	1989	226.5	C6 (Tetra)	163/240
P05	1956	2007	2.5	C5 (Tetra)	186/210
P06	1960	1982	38.9	Th5 (Para)	223/240
P07	1949	2007	4.2	C6 (Tetra)	155/180
P08	1959	1979	341.7	Th11 (Para)	199/240
P09	1943	2007	11.9	Th6 (Para)	149/210
P010	1949	1975	394.6	Th8 (Para)	113/210
P011	1966	2007	5.5	Th4 (Para)	161/180

P012	1992	2008	1.6	Th12 (Para)	165/210
P013	1963	2005	33.2	C7 (Tetra)	157/210
P014	1965	2007	6.8	C6 (Tetra)	159/240
P015	1984	2006	22.1	L1 (Para)	190/210

Mean	1965.53	1998.93	86.05		
Median	1963	2007	11.9		
SD	14.83	12.08	134.42		

**Table 2:** Classification accuracy (%) of the maximal peak and its latency (delay) after cue onset for all 15 patients and all combinations. Accuracies in bold differ significantly from chance level according to the number of trials (cf. [13]).

Patient	Left versus right hand		Left hand versus feet		Right hand versus feet	
	Accuracy (%)	Delay (s)	Accuracy (%)	Delay (s)	Accuracy (%)	Delay (s)
P01	56.2	3.5	54.8	4.5	56.9	1.0
P02	**78.1**	2.0	**86.8**	2.0	**83.1**	3.0
P03	**63.5**	1.0	**62**	1.5	**63.3**	1.5
P04	**63.5**	1.5	**62.3**	2.0	**67.7**	1.5
P05	58.7	2.5	56.2	1.5	51.4	4.5
P06	57.9	1.5	**65.0**	2.5	58.5	2.5
P07	57.2	2.0	**64.3**	1.5	55.9	2.0
P08	**60.4**	1.5	**63.6**	2.0	**62.0**	1.5
P09	**83**	1.5	**86.3**	1.5	**71.1**	2.5
P010	**68.1**	2.0	**85.7**	2.0	**78.8**	2.0
P011	**76**	1.5	**77.7**	1.5	**75.2**	1.5

P012	59.8	2.0	**70.6**	1.5	**63.1**	0.5
P013	57.1	2.5	54.4	4.0	56.8	0.5
P014	**64**	0.0	**65.2**	3.0	60.1	3.5
P015	**71.8**	2.0	**67.9**	2.5	**71.8**	2.5

Mean	65.02	1.8	68.19	2.23	65.05	2.03
Median	63.5	2	65	2	63.1	2
SD	8.52	0.77	11.08	0.94	9.24	1.09

**Table 3:** Mean classification accuracy for tetraplegic and paraplegic patients.

Motor imagery	Spinal cord injury			
	Paraplegic		Tetraplegic	
	Mean	SD	Mean	SD
L versus R (%)	67.56	8.86	62.13	7.7
L versus F (%)	72.35	9.72	63.43	11.25
R versus F (%)	67.99	7.23	61.70	10.67
